# Towards integrated care in breastfeeding support: a cross-sectional survey of practitioners’ perspectives

**DOI:** 10.1186/s13006-016-0072-y

**Published:** 2016-06-03

**Authors:** Stefanie Inge Rosin, Irena Zakarija-Grković

**Affiliations:** www.stillberatung-rosin.de, Berlin, Germany; School of Medicine, University of Split, Split, Croatia

**Keywords:** Integrated care, Breastfeeding support, Practitioners in breastfeeding support, Continuum of care, Consistency of care

## Abstract

**Background:**

Integrated care is defined as concerted action of healthcare providers ensuring continuity of care within a patient-centered approach, thus contributing to healthcare efficiency and quality. Apart from the WHO/UNICEF Baby-Friendly Initiatives, integrated care has been poorly explored within the context of breastfeeding support. The aim of this study was to investigate the experience of breastfeeding support practitioners, identifying barriers and facilitators towards integrated care.

**Methods:**

A 62-item survey was conducted among 900 participants at 3 international breastfeeding conferences. Analysis included uni-and bivariate descriptive statistics, categorizing of mutually exclusive response groups and thematic networks analysis of responses to 18 open-ended items.

**Results:**

Three-hundred-and-one participants (33 % response), from 34 predominantly industrialized countries (98 %) on nearly all continents, responded to the survey. Norwegian residents alone, felt sufficiently supported in providing breastfeeding support by other healthcare providers, the work environment, society, the media and their National Breastfeeding Committee (*P* < 0.05). Out of 11 suggested measures for effective breastfeeding promotion, 96 % of respondents ranked integrated care as the most important. The largest response group identified in open-ended items, as a major barrier to integrated care in breastfeeding support, was “lacking or failing health promotion strategies” (*n* = 454), followed by “a lack of vertically integrated care” (*n* =268), described mainly as unsatisfactory cooperation within healthcare. This inconsistency of care also impairs “shared decision-making” on infant feeding for parents, including accessibility of information and support (*n* = 265). Among other measures, 29 % of respondents recommended incentivizing integrated breastfeeding support within healthcare. Two figures, based on open-ended response evaluations, illustrate participants’ ideas of the National Breastfeeding Committees’ role in coordinating policies and protagonists towards integrated breastfeeding support, and a family-centered model of integrated care to facilitate successful breastfeeding.

**Conclusions:**

According to practitioners in breastfeeding support, integrated care is essential for successful breastfeeding. Quality and accessibility of breastfeeding support should be motivated by healthcare system incentives, to counter the reported lack of consistency of care within and beyond healthcare. To effectively integrate a continuum of breastfeeding support into healthcare and society, a policy consensus and strong political action are indispensable, with coordination by an empowered National Breastfeeding Committee.

**Electronic supplementary material:**

The online version of this article (doi:10.1186/s13006-016-0072-y) contains supplementary material, which is available to authorized users.

## Background

Integrated care can be described as a collaborative approach among healthcare professionals. The World Health Organization (WHO) defines integrated care as [1]:

“the management and delivery of health services so that clients receive a continuum of preventive and curative services, according to their needs over time and across different levels of the health system.”

Integrated care has become increasingly important for healthcare systems to optimize continuity, consistency and quality of care, while ensuring interdisciplinary co-operation and cost-efficiency.

Integrated care requires consistency of treatment and advice from multi-professional healthcare providers, independent of setting, otherwise:

“Without integration at various levels, all aspects of health care performance suffer. Patients get lost, needed services fail to be delivered, or are delayed, quality and patient satisfaction decline, and the potential for cost-effectiveness diminishes” ([2], page 2).

Adequate education, exchange between healthcare providers and respect of different healthcare disciplines are indispensable for integrating care toward a patient-centered focus, optimizing cost-efficiency, patient satisfaction and health outcomes.

Breastfeeding rates dropped to an all-time low in the mid-20th century [3] as a result of industrialization [4] and medicalization of birth and infant feeding [5]. Today, breastfeeding rates are increasingly considered core health outcomes for maternal and child health [6–8] and infant survival [9, 10]. Improved breastfeeding rates support cost savings in terms of disease prevention and health promotion [10, 11].

Establishing and sustaining a breastfeeding relationship is a vulnerable process, which has to be learned by the mother-baby-dyad [12]. Support for this relationship involves an array of health workers and services through the ante-, peri- and postnatal period. Consequently, WHO and UNICEF developed the Baby-Friendly Initiatives [13], aimed at integrating breastfeeding support into key maternity healthcare settings including hospitals [14] and the community [15]. Hospital accreditation requires following the evidence-based “Ten Steps to successful breastfeeding”, representing an integrated care pathway of breastfeeding support before, during and after hospital admission. This includes, among other quality criteria, providing information to parents during pregnancy, skillfully supporting bonding and latching, avoidance of mother-baby separation and providing mother support groups and/or hotlines after hospital discharge.

Furthermore, WHO and UNICEF recommend the creation of National Breastfeeding Committees (NBCs) to protect, promote and support breastfeeding [16]. Foundations of NBCs started in the 1990s following the Innocenti Declaration, for instance in Canada, USA and Poland, later in Greece, Sweden, Switzerland, Croatia and Romania; while some are no longer active, e.g. in Romania, or have never been founded, e.g. in Japan or France ([17, 18], e-mail correspondence of the first author with governments and practitioners in breastfeeding support in January and February 2016). Their original tasks include the prevention of unethical marketing of breast-milk substitutes according to the International Code [19], the creation of breastfeeding-friendly legislation and policies, including adequate maternal leave, and the spreading of the Baby-Friendly Initiatives on a national level [13–16]. These policies are part of the “Global Strategy for Infant and Young Child Feeding” (Global Strategy), which aims to re-establish breastfeeding as the universal infant feeding norm [20].

One obstacle to achieving this goal is the documented lack of healthcare professionals trained in providing breastfeeding support [21–24]. Consequently, as a key measure in achieving Baby-Friendly standards, WHO and UNICEF mandate training for all maternity healthcare providers. To further compensate this shortage, a new cadre of dedicated practitioners in breastfeeding support has emerged, initially as volunteers including La Leche League International counsellors, who provide direct assistance to mother-baby dyads and facilitate mother-to-mother support groups [25], followed by other NGOs with the same focus [26, 27]; then as healthcare professionals, including International Board Certified Lactation Consultants (IBCLCs) [28] and physicians with a special interest in breastfeeding medicine [29]. All of these practitioners in breastfeeding support play an important role in breastfeeding initiation and sustainment, implementation of Baby-Friendly standards, increasing breastfeeding rates, and improving mothers’ satisfaction with healthcare [30–32]. Midwives also perform a vital role in breastfeeding support, especially when facilitating natural childbirth and breastfeeding initiation within the first hour after birth [33, 34].

This study investigates the integrated care concept within breastfeeding support, by analyzing and describing from the perspective of practitioners, how breastfeeding support functions within the following integrated care fields, and how it can be improved [35]:

- Vertical integration defined across primary, hospital and tertiary care services
- Integration within one sector (e.g. within maternity care services)
- The use of system incentives, such as governance, guidance, funding and payment mechanisms, that seek to embed and reward integrated care
- Implementation of health promotion strategies
- The impact of integrated care in reducing health inequalities
- Horizontal integration between health services, social services and other care providers
- Delivery systems that bring together clinicians and managers, funders and deliverers, professionals and patients.
- Integration between care providers and patients that supports shared-decision making

## Methods

### Study design and setting

This cross-sectional survey was conducted in 2008 at 3 major international breastfeeding conferences: European Lactation Consultants Alliance (ELACTA, formerly VELB = Verband Europaeischer LaktationsberaterInnen [Association of European Lactation Consultants])/International Lactation Consultant Association (ILCA) Conference, October 1–3; Academy of Breastfeeding Medicine (ABM) regional meeting, October 4–6, both in Vienna, Austria; and La Leche League Germany National Meeting (LLL), September 26–28, Dassel, Germany.

### Questionnaire design

The questionnaire was created in the context of a PhD program undertaken in the Faculty of Health Sciences at the University of Bielefeld, Germany, using relevant literature on questionnaire design [36–39]. Feedback was provided by researchers from the Leibniz Institute for the Social Sciences “GESIS”, Mannheim, Germany.

The survey consisted of 62 questions (44 closed, 18 open), covering a range of topics, including: respondent profile (11 items), work situation (29 items), contentedness (3 items), priority measures for the integration of breastfeeding support (11 items), expectations (3 items), and future prospects (5 items). A four-response Likert scale provided 2 levels of agreement or importance of the item’s statement, and 2 levels of disagreement or unimportance, without a neutral response possibility. Several open questions were provided to enable respondents to elaborate upon closed question topics.

The questionnaire was first developed in German and then translated into English, to be merged into a bilingual questionnaire. It was pilot tested among a group of 12 practitioners in breastfeeding support to assess intelligibility, clarity and relevance. Subsequently, the questionnaire was shortened, clarified and re-structured. Native speakers in German and English, and public health scientists approved the final version. Internal consistency was acceptable with a Cronbach’s Alpha of 0.7. The complete questionnaire is available in the dissertation annex [40].

### Data collection and analysis

The survey was distributed to conference participants, who returned the completed form to a collection point at the conference venues or by postal mail. Eighteen respondents were contacted by e-mail in a second round to further clarify responses and several missing values. German open-ended responses were translated into English by the first author, who has a university degree in translation and is a German native speaker, and finally checked by an English native speaker, who is knowledgeable in the field of lactation.

To enable cross tabulations with selected characteristics, new variables with 2 categories were formed within the statistical database, to compare the chosen characteristic with the rest of the sample (e.g. the Norwegian residency was coded as 1, all other residence countries were coded as 2). Thus outcomes were cross tabulated using chi-square tests and exact Fisher tests by age quartiles, profession, residence (for the top 12 represented countries), type of conference attended, voluntary or paid breastfeeding support, quantity of breastfeeding support provided, estimate of compensation, acknowledgement on the job, and degree of personal experience with breastfeeding. Statistically significant associations were set at a level of *P < 0.05*, based on a four-response Likert scale. Statistical analysis was carried out using SPSS software (version 17, Chicago, IL, 2008).

To further explore the importance respondents ascribed to “integrated care” for effective breastfeeding support, we evaluated open-ended responses using qualitative methods. Practitioners in breastfeeding support provided free-text responses to most open-ended items, with responses ranging from a few words to complete sentences. These free-text responses were read multiple times and discussed within the research team to elicit their integrated care relevance and be sorted into integrated care categories, using a deductive approach. Categorization followed the above mentioned integrated care fields. Thus mutually exclusive categories were developed and quantified in groups and sub-groups, using Excel computer software [41]. Where useful, direct quotes are presented to illustrate the categorizations.

Since we merged two complex topics “integrated care” and “breastfeeding support”, we aimed at presenting this complexity in an understandable way. Therefore we further applied thematic networks analysis, which allowed a rich description of the large data set of open-ended responses at different organizational levels [42]. We indexed respondents’ statements into categories using an inductive approach, thus establishing a framework of thematic ideas of basic, organizing and global themes. This process included several rounds of discussion, reviews and revisions within the team, finally reaching consensus. To optimize comprehensibility of the complex systemizations detected, results of thematic networks analysis are presented diagramatically.

### Ethical approval

All organizations involved gave permission for the study. Participants were informed that the questionnaire was to be used for a dissertation study in public health and that participation was voluntary and anonymous, with the option of providing contact details. A completed and returned questionnaire was interpreted as an indication of consent. Ethical approval for the dissertation study was obtained from the ethical committee of Bielefeld University (No. #2013-001), where the study was conducted within a PhD program in health sciences.

## Results

### Participants

Of the 900 questionnaires distributed 301 were returned, representing a response of 33 %. The majority were VELB/ILCA conference participants (77 %), 15 % were from LLL Germany and 8 % from the ABM conference. Respondents came from almost every continent and thus represented an international sample, with the exception of South America. There was a clear prevalence of participants from industrialized countries (98 %). Respondents were between 21 and 78 years old and included 3 male participants. Eighty-five percent of respondents claimed to have personal breastfeeding experience (*n* = 256), with 72 % of those over one year per child (*n* = 183). Ninety percent of survey respondents had a qualification in breastfeeding counselling (*n* = 272), with the ratio of unpaid to paid practitioners in breastfeeding support being roughly 1:3 (Table 1).

**Table 1:** Characteristics of respondents (*n* = 301)

Characteristic	*n* (%*)*
Continent of residence	
Europe	244 (81)
North America	28 (9.3)
Oceania	18 (6.0)
Asia	9 (3.0)
Africa	2 (0.7)
Top twelve represented countries	
Germany	107 (35.5)
Austria	44 (14.6)
Switzerland	26 (8.6)
USA	21 (7.0)
Australia	16 (5.3)
Netherlands	15 (5.0)
Italy	8 (2.7)
Canada	7 (2.3)
Belgium	6 (2.0)
Luxembourg	4 (1.3)
Norway	4 (1.3)
Sweden	4 (1.3)
Profession^a^	
Nurse	146 (48.5)
Pediatric	52 (17.3)
Maternity	47 (15.6)
Lactation Consultant	26 (8.6)
General	21 (7.0)
Midwife	53 (17.6)
Certified Doula	1 (0.3)
Physician	44 (14.6)
Pediatrician	25 (8.3)
Gynecologist	11 (3.6)
General Practitioner	9 (3.0)
Researcher (field of research as stated by respondents)	19 (6.3)
Medical	7 (2.3)
Public health	7 (2.3)
Others^b^	5 (1.7)
Public health official	39 (13.0)
Trainer of health care providers	16 (5.3)
Social and health service provider	10 (3.3)
Infant nutritionist	7 (2.3)
Baby-Friendly Hospital Initiative coordinator	2 (0.7)
Others^c^	7 (2.3)
Health policy maker	8 (2.6)
Advocator for breastfeeding	3 (1.0)
Government advisor	2 (0.7)
Others^d^	3 (1.0)
Professions outside the health care sector^e^	28 (9.3)
Breastfeeding support qualifications^f^	327
International Board Certified Lactation Consultant	227 (75.4)
Voluntary^g^	73 (24.3)
Other ^h^	27 (9)
Payment for breastfeeding support	
Unpaid	66 (22)
Unpaid and paid	23 (7.6)
Paid	194 (64.5)

^a^Exceeds 100 % due to multiple professions of individual respondents, while 3 respondents reported no qualification

^b^Natural sciences, Psychology, Political Sciences, Social Sciences, Epidemiology

^c^National Breastfeeding Committee member, nutritionist, supporter of health care providers, prevention activist

^d^Local public health department, Health Ministry, National and international policies

^e^Teacher, psychologist, administrator, bank clerk, parents’ counsellor, bookseller, lecturer, translator, TV journalist, student, optician, dental hygienist, consultant for medical products, physiotherapist, speech therapist, bio-medical analyst

^f^Exceeds 100 % due to multiple qualifications: IBCLC + LLL 7.6 % (*n* = 23), IBCLC + AFS 1 % (*n* = 3), IBCLC + ABA 1 % (*n* = 3)

^g^La Leche League 21 % (*n* =64), Arbeitsgemeinschaft Freier Stillgruppen 2 % (*n* = 6), Australian Breastfeeding Association 1 % (*n* = 3)

^h^Health care providers 8 % (*n* = 24), Breastfeeding mothers 7 % (*n* = 2), WHO 40 h course qualification 0.3 % (*n* = 1)

There were only a few significant differences by sociodemographic characteristics between response groups; these differences are reported in the results section, where relevant. Overall the response pattern within the quantitative items was predominantly homogenous, indicating extensive consensus among our international and interdisciplinary sample.

### Integrated care in breastfeeding support

Overall, respondents lamented a lack of consistency and continuity of breastfeeding support, stating that their service was insufficiently supported. Only Norwegian residents felt sufficiently supported in providing breastfeeding support by other healthcare providers (*p* = 0.01), the work environment (*P* = 0.01), society (*p* = 0.009), the media (*p* = 0.005) and their National Breastfeeding Committee, due to their successful promotion of breastfeeding (*p* = 0.000). Consequently, when ranking 11 measures for breastfeeding promotion, respondents chose “integrated care” as the most important (Table 2). Based on the relevant integrated care fields listed in the background section, Table 3 presents an overview of the barriers to integrated care in breastfeeding support including quantifications, using deductive evaluations of open-ended responses. Further details on the largest response groups “vertically integrated care” and “health promotion strategies” can be found in Additional files 1 and 2.

**Table 2:** Priority ranking of eleven suggested measures for effective breastfeeding promotion

Univariate statistical evaluations		4-point Likert scale			
Mean score	Measure for breastfeeding promotion	very important (4^*a*^) *n*/%	important (3^*a*^) *n*/%	less important^*b*^(2^*a*^) *n*/%	not at all important^*b*^(1^*a*^) *n*/%
3.87	Integrated care within breastfeeding support	256/85.0	34/11.3	1/0.3	1/0.3
3.86	Promotion of breastfeeding integrated into health policies	252/83.7	37/12.3	2/0.7	-
3.75	Education of the public on benefits of breastfeeding and risks of substitutes	225/74.8	57/18.9	9/3.0	-
3.74	Promotion of research independent of commercial sponsoring	219/72.8	64/21.3	5/1.7	-
3.73	Media campaigns for breastfeeding	219/72.8	61/20.3	9/3.0	-
3.70	Implementation of the International Code of Marketing of Breast-milk Substitutes into legislation	220/73.1	56/18.6	9/3.0	4/1.3
3.61	Implementation of Baby-Friendly standards as the norm	201/66.8	65/21.6	22/7.3	1/0.3
3.59	Upgrade the profession lactation consultant to create career possibilities	188/62.5	83/27.6	18/6.0	-
3.58	Implement the IBCLC credential as educational standard within healthcare	193/64.1	78/25.9	17/5.6	3/1.0
3.57	Governmental monitoring of and penalty for Code violations	180/59.8	91/30.2	9/3.0	4/1.3
3.38	Development of a human milk bank network	133/44.2	110/36.5	25/8.3	3/1.0

^*a*^Statistical value for SPSS evaluations

^*b*^Conspicuous minority was addressed in a second question round, and their arguments were discussed

**Table 3:** Barriers to integrated care in breastfeeding support, according to integrated care fields

Integrated care response groups according to categorization of open-ended responses (n)					**Total** ***n*** = **1**,**168**		
Vertical integration^a^ (268)	Within one sector (52)	Incentives (87)	Health promotion strategies^b^ (454)		Health inequalities (19)	Horizontal integration (23)	Shared decision- making (265)
Lack of concerted action within healthcare to cooperate towards integrated care in breastfeeding support (88) Lack of healthcare providers competent in breastfeeding support, lacking recognition of expertise within healthcare (64) Lack of physicians’ cooperation towards integrated care in breastfeeding support (49) Lack of researchers’ knowledge on breastfeeding, lack of practice-oriented research to improve breastfeeding support (34) Lack of cooperation between voluntary and professional practitioners (19) Lack of human milk bank networks to facilitate integrated care in breastfeeding support (14)	Lacking implementation of Baby-Friendly standards (26) Lacking quality of breastfeeding support within hospitals (14) Lacking integration of adequate breastfeeding support into routine hospital care (12)	Lacking incentives of health insurance companies to motivate breastfeeding as disease prevention (35) Lack of healthcare system incentives to prevent unnecessary supplementation and interventions at birth (24) Lack of adequate compensation from health insurance companies for receiving and providing breastfeeding support (16) Lacking incentives for parents for breastfeeding and the donation of human milk (12)	Lacking policies and their implementation to protect, promote and support breastfeeding (127) Lacking impact of NBCs on policies and lacking coordination of policies and protagonists (76) Lack of high-quality and ethically sound research and its funding, independent of commercial interests (68) Lacking support and funding for breastfeeding promotion from governments, health insurance companies, politicians (42) Lacking implementation and monitoring of the Code^**d**^ (36)	Lacking research on policy implementation (26) Lacking promotion of breastfeeding as a preventive measure (25)	Differing breastfeeding rates among social classes contribute to exacerbate health inequalities (11) Lacking access to adequate breastfeeding support, independent of socioeconomic factors (4) Lacking access to breastfeeding support impairs patient satisfaction with healthcare (4)	Lacking education of kindergarten teachers and lacking normalization of breast-feeding in child education (13) Lacking competence of school teachers in the field of breastfeeding (5) Lack of family counselling services with competence in breastfeeding support (5)	Lack of visible marketing strategies for breastfeeding to counter formula marketing (93) Lacking perception of breastfeeding as the norm, and lacking breastfeeding-friendliness in society (57) Lack of consistent information on breastfeeding by healthcare professionals (35) Lacking dissemination of relevant research to practitioners and the public (35) Lacking information and education of the public, including prenatal courses (34) Lacking control of sponsored media portrayal of infant feeding (11)
				Lacking foundation of NBCs ^**c**, **d**^ as Delivery Systems in several countries (20)			
				Lacking legislation to protect and promote breastfeeding, including adequate maternal leave (20)			
				Lack of health policies facilitating a patient-centered approach in providing breastfeeding support (8)			
				Lack of prioritizing breastfeeding protection and promotion towards “health before profit” (8)			

^a^More details of the “Vertical Integration” category can be found in Additional file 1

^b^More details of the “Health Promotion Strategies” category can be found in Additional file 2

^c^More details of the “Delivery System” category can be found in Fig. 1

^d^See “Abbreviations” following the main manuscript

### Vertically integrated care

Vertically integrated care refers to optimal collaboration between providers at different levels, such as primary, secondary and tertiary care.

Eighty-nine percent of respondents reported a failure of vertical cooperation in their work environments, attributing this to a lack of knowledge and skill in providing breastfeeding support among healthcare providers, especially physicians (Table 3). Support and acknowledgement by other healthcare professionals, including superiors, was also described as poor with only 7.6 % of respondents reporting a supportive work environment. Additional file 1 shows in detail the barriers against vertically integrated breastfeeding support, described in open-ended responses from the perspective of practitioners in breastfeeding support. This includes interesting aspects and quotations mentioned within response groups, while each quotation goes with the respondent’s residence country, the profession and qualification in breastfeeding support.

When asked how important the education of healthcare professionals in breastfeeding support to IBCLC standard was, this measure was considered as very important/important by 90 % (Table 2), with almost all participants agreeing that the IBCLC credential should become the standard for all healthcare providers in maternity care to facilitate vertically integrated breastfeeding support. Several respondents suggested that healthcare professionals other than maternity care providers should be enabled to provide basic support for breastfeeding, while all healthcare providers should become aware of lactation consulting possibilities, including timely referrals to lactation consultants (12.9 %; *n* = 39). Ninety-four percent of nurses/midwives (*n* = 187), 88 % of physicians (*n* = 39) and 92 % of other professionals (*n* = 53) thought that those working with breastfeeding families should be trained as IBCLCs. However, the IBCLC qualification was considered important by only 50 % of residents from Norway (*p = 0.03*), Sweden (*p =* 0.02), Belgium (*p* = 0.03) and Canada (*p* = 0.02).

Respondents called for the integration of voluntary and professional breastfeeding support services and improved collaboration between them (6% / Additional file 1), with one respondent requiring:

*“Easy access to mother support groups”*

(Netherlands/General Practitioner/Healthcare Provider)

Additionally, respondents considered the extension of a human milk bank network as an important resource for providing human milk to infants in need through the cooperation of different care levels (80.7 %; *n* = 243; Table 2).

### Integrated care within one sector

The setting of interest for this sector is the maternity hospital. Integration of care in this setting was considered possible if hospitals were to achieve Baby-Friendly designation, which was considered very important/important by 88 % (Table 2). Those who considered it as less important (7.6 %/*n* = 23) stated that it was not achievable in their setting, or cited the misuse of the title with no real fulfillment of the standards, especially in Romania and USA. Only 3 % of lactation professionals (*n* = 10) explicitly reported a supportive work environment in the hospital setting.

Seventeen percent of participants described difficulty implementing evidence-based breastfeeding support into hospital care (Table 3), including slow progress and frequent backlash. Important aspects mentioned within this category are the lack of remuneration and acknowledgement for their work in this setting, lack of time for lactation consulting, lack of staff and breastfeeding clinics for integrated care within this setting and after hospital discharge, adverse routines such as the routine supplementation with infant formula,the separation of mothers and infants, and the failure of collaboration with non-educated healthcare staff with non-supportive attitudes. Two participants wrote:

“ *Currently the development on my ward towards improved breastfeeding support is stagnating*” (Luxembourg/Pediatric Nurse at Maternity Ward/IBCLC)*“*

*“The routines in my hospital prevent the application of my knowledge and skills as a lactation consultant.”* (Germany/Midwife/IBCLC)

### Incentives for breastfeeding support within the healthcare system

Incentives are defined as governance, guidance, funding and payment mechanisms that seek to embed and reward integrated care. Twenty-nine percent of respondents called for the integration of breastfeeding support into health services by establishing system incentives (Table 3).

Within the “incentives” category, several respondents advocated for the recognition of midwifery and breastfeeding support as essential healthcare, complaining about non-supportive and counter-productive structures in the current healthcare systems both for lactation professionals and midwives, thus impairing natural birth and breastfeeding (5 %). Two respondents wrote:

*“Abolish financial incentives for birth interventions and the use of formula, such as formerly within the US Women, Infant and Children Program…”*

(USA/Maternity Nurse/IBCLC)

“ *Provide regular remuneration from health insurance companies for breastfeeding support*” (Poland/General Practitioner/IBCLC)

Four percent of participants suggested offering parents incentives for successful initiation and sustainment of breastfeeding, such as free lactation consulting services, equal access to breastfeeding support and remuneration for the donation of human milk. Ten percent of respondents defined the establishment of incentives for natural birth and breastfeeding, both for families and for the healthcare system, as a key role of health insurance companies (Table 3). They should acknowledge breastfeeding as a preventive measure deserving of financial incentives and adequate remuneration (7 %). Furthermore, health insurance companies should play an active role in the prevention of unnecessary interventions at childbirth and unnecessary supplementing with infant formula (3 %). Several respondents suggested that the National Breastfeeding Committee should lobby for this to occur (see section “delivery system”). One respondent wrote:

*“There will be no increase in breastfeeding rates in the near future, as long as the current trend of obstetric interventions during childbirth continues.”*

(Austria/Maternity Nurse/IBCLC)

### Health promotion strategies towards integrated care within breastfeeding support

Out of 11 measures, 96 % of participants ranked breastfeeding promotion integrated into national health policy second in order of importance (Table 2). One open-ended question directly asked expectations of respondents from health policies. However, responses in the “health promotion strategies” category exceeded the sample size (*n* = 301), since remarks on health policies were provided in several items throughout the questionnaire (*n* = 454). This high response confirms the major importance given by practitioners in breastfeeding support to policies that support breastfeeding (Additional file 2). While slightly less than half of the respondents stated national policies promoting breastfeeding exist in their country of residence (46.6 % versus 53.4 % with no policies), only a quarter qualified their implementation as successful (25.1 % versus 74.9 % unsuccessful). Hence, several respondents suggested that breastfeeding protection and promotion should become a priority in health policies, prioritizing *“health before profit”* (Additional file 2), while one complained about

*“the slow and unsupported progress of a breastfeeding culture”*

(Australia/Midwife/IBCLC).

Implementation of the International Code of Marketing of Breast-milk Substitutes into legislation was considered as an important measure by 91.7 % of respondents (Table 2). Four percent of respondents considered this measure less important and justified this by wishing for less governmental regulations in general; an assumption that producers of breast milk substitutes will always find loopholes to get around laws and fearing that restriction of advertising might be seen as limiting freedom of expression. Ninety percent of respondents considered monitoring of, and penalty for violations of the Code very important or important, while 4 % considered it less or not at all important (Table 2). Reasons for considering this measure less important were a belief that it is not practicable or feasible to control the food industry and that governments have acted as major formula buyers in the past, e.g. in the US Women, Infant and Children program, which would be hard to overcome.

### Health inequalities in providing breastfeeding support

Six percent of respondents expressed concern for inequalities in healthcare because of the lack of access to breastfeeding support for families of lower socio-economic status (Table 3). One feared that

*“the gap between well-educated families of higher socioeconomic status with higher breastfeeding rates, and lower breastfeeding rates of disadvantaged families, will worsen in the future”* (Germany/Bank clerk/LLL).

In this category, respondents called

*“the increasing rate of teenage mothers problematic”*

(USA/Maternity Nurse/IBCLC);

while several respondents feared that a two-class society will develop, mentioning

*“the challenge to support the vulnerable”* (Croatia/General practitioner/IBCLC).

Consequently, several respondents requested equal access to breastfeeding support for all families (Table 3) to be enforced by health policies (Additional file 2). Two respondents wrote:

*“Integrate lactation consulting into the national healthcare system”*

(China/Obstetrician-Gynecologist/IBCLC + LLL)

and guarantee

*“lactation consulting for everyone”*

(Germany/Pediatric nurse/IBCLC).

### Horizontally integrated care

Horizontally integrated care is defined as care between health services, social services and other care providers. Seven percent of respondents emphasized the importance of horizontal cooperation with and between education providers, including school teachers, kindergarten teachers, day care nurses, social workers and psychologists, who should be educated in breastfeeding support (Table 3). They advocated for the education of children starting at kindergarten with the aim of normalizing breastfeeding e.g. by portraying it in children’s books (5 %). In addition, several respondents suggested a network of family counselling centers that offer breastfeeding support (1.7 %/Table 3), with 3 respondents suggesting:

“*The Lactational Amenorrhea Method should be included in family planning counseling*” (USA/Pediatrician, Preventive Medicine, Epidemiology, Maternal and Child Health, State and National and International Health Policies/FABM + IBCLC + LLL)

*Teach breastfeeding as normal from kindergarten age*”

(USA/Lactation Consultant/IBCLC + LLL)

*“Educate the importance of breastfeeding at school”*

(Japan/Pediatrician/IBCLC)

### Delivery system for integrated care in breastfeeding support

A delivery system for integrated care should bring together clinicians and managers, funders and providers, professionals and patients. Respondents felt that the National Breastfeeding Committee bears the responsibility for such integration as part of its tasks to protect, promote and support breastfeeding (75.4 %/*n* = 227). Eighty-one percent of respondents were resident in countries with an established NBC in 2008, including nearly all of the top twelve represented countries (Table 1), with the exception of Australia and The Netherlands. The remaining 19 % of respondents had no NBC established in their residence countries by 2008, including China, UAE, Japan, Lithuania, Bosnia-Herzegovina, Spain and others ([18, 19], e-mail correspondence of the first author with practitioners in breastfeeding support in 2016). Respondents were ambivalent in their assessment regarding whether their National Breastfeeding Committee was promoting breastfeeding successfully (52.3 % successful versus. 47.7 % unsuccessful), with the exception of Norwegian respondents, who assessed the performance of their “National Advisory Unit” as successful, compared to all other respondents (*P* = 0.000). When asked about their expectations of the Committee, participants expected it to play a key role in re-establishing a breastfeeding culture and to act as a major contact point for all practitioners, researchers, policy makers and parents.

**Fig. 1:**
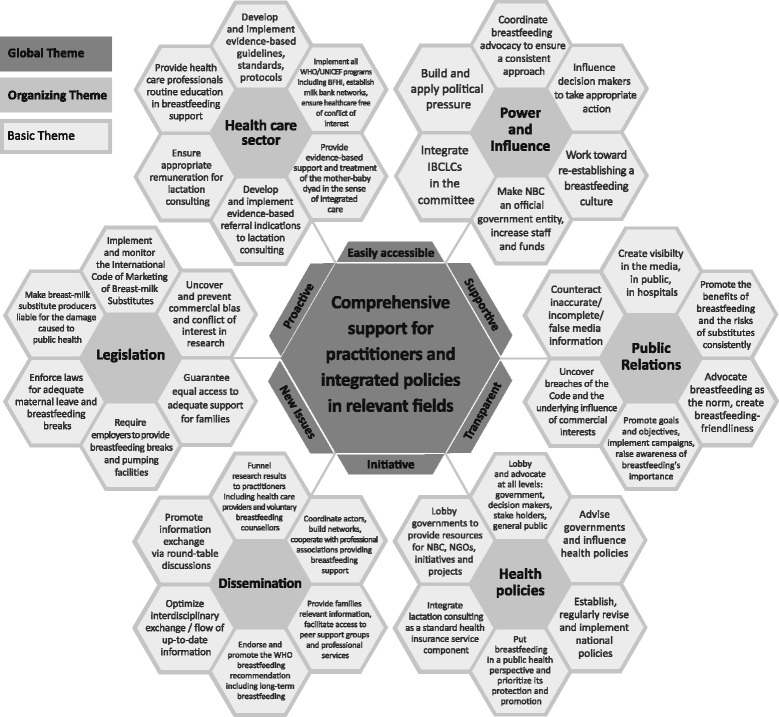
What practitioners in breastfeeding support expect from the National Breastfeeding Committee

The comprehensive tasks of the National Breastfeeding Committee, as identified by respondents, are shown in Fig. 1. The figure reflects practitioners’ expectations from the National Breastfeeding Committee, regardless of whether an NBC was already established in their residence countries or not, with both groups showing equal response rates of about 55 %. Keywords provided by respondents were first analyzed into the global theme, which is shown in the center of Fig. 1, together with desired attributes of the NBC. The organizing themes are the fields of action practitioners expect the NBC to take, while basic themes show details of the desired activities. After having identified the themes, we refined the wording of Fig. 1’s keywords, with the aim of optimally representing the many important tasks described by respondents. To successfully fulfill the defined major tasks, several respondents recognized the need to provide the National Breastfeeding Committee with adequate funds, staff and authority, while some lamented that a National Breastfeeding Committee was not yet founded in their countries (e.g. Australia, France, Israel, Netherlands), with one respondent stating:

*“We have a new Committee of Breastfeeding in Finland, I’m sure something will happen…”* (Finland/Public Health Nurse, Head Nurse/Healthcare Provider)

In reply to the open-ended question, what they expect from the NBC, two respondents having no NBC in their residence country wrote:

*“A strong policy that is supported by appropriate legislation to give adequate maternity leave and breastfeeding breaks.”* (UAE/Midwife/IBCLC)

*“NBC should advise government on legislation to support breastfeeding.*” (UK/Midwife/IBCLC)

Two respondents having an NBC in their residence country wrote:

*“The NBC should plan and coordinate a national policy to be implemented with political support”* (Italy/Pediatrician/Healthcare Provider)

*“What can I expect from a committee without funds, power and influence?”*

(Germany/Midwife/IBCLC)

Respondents considered the original tasks of the NBC, as defined in 1990 [16], to be entirely relevant today (Fig. 1). Additionally, several respondents suggested the planning and conducting of a marketing strategy to convey a positive message on breastfeeding for increased public support, with the goal of making society more breastfeeding-friendly. Ninety-three percent of respondents considered advertising campaigns as very important or important (Table 2). Two respondents wrote:

*“Progress is only possible with broad campaigns and large investments.”*

(Germany/Pediatric Nurse/IBCLC)

*“Create an image of breastfeeding as smart, cool and career-right.”*

(Norway/Gynecologist, Government Advisor/Healthcare Provider)

### Decision making on infant feeding

Eighty-eight percent of respondents reported manifold obstacles on different societal levels for parents in making a shared decision on infant feeding (Table 3). The main obstacle was considered to be inconsistent, incorrect, outdated and non-evidence-based information provided by healthcare professionals to families, making parents insecure and undermining breastfeeding success, while two respondents lamented:

*“Breastfeeding myths from healthcare providers and aged people”*

(Taiwan R.O.C./Obstetrician,Gynecologist/IBCLC + LLL)

*“Too many different opinions”* (Switzerland/Nurse/IBCLC)

Within this category, several respondents called for consistent, evidence-based, up-to-date and unequivocal information on infant feeding to facilitate breastfeeding and empower the family, with one respondent suggesting:

*“Antenatal classes which include family members (father, aunts, grandparents)”*

(New Zealand/Maternity Nurse/IBCLC)

In this context, several respondents requested a clear commitment by governments to the WHO public health recommendation of exclusive breastfeeding for the first 6 months and continued breastfeeding beyond 1 year of age. Twelve percent of respondents also highlighted the lack of dissemination of relevant research to back up practitioners and families, and thus provide the public with up-to-date and evidence-based facts. Eleven percent called for independent research, free from commercial interests to avoid commercial bias (Table 3). Ninety-four percent considered the promotion of independent research as a very important/important task (Table 2).

While 52 % of practitioners in breastfeeding support considered the media as non-supportive, 4 % further explained in open-ended responses that parents are influenced by negative media reports harming the image of breastfeeding in society, providing incorrect information and aggressively marketing breast-milk substitutes. One respondent wrote:

*“Ads of breast milk substitutes are rampant on mass media”*

(South Korea/Pediatrician/Healthcare provider)

To promote shared decision-making on infant feeding unbiased by commercial interests, several respondents suggested that the National Breastfeeding Committee should counteract incorrect statements in the media and thus counter the continued attempt of the formula industry to establish bottle-feeding as the norm for infant feeding (Fig. 1). While 47 % of respondents lamented lacking societal support in general, 11 % further explained this shortcoming in open-ended responses, mentioning that the parents’ social environment often provides incorrect information on infant feeding. This resulted in parents being subject to many different opinions including breastfeeding myths, making them feel insecure and impairing breastfeeding success. One respondent wrote:

*“There is a cultural and educational ignorance of the natural breast function.”*

(USA/Maternity Nurse/IBCLC)

In this context, the education of the public about the benefits of breastfeeding and risks of substitutes scored 94 % “important” versus 3 % “less important” (Table 2). One respondent who considered this measure as less important justified this by stating that:

*“Without consistent breastfeeding support from healthcare providers, the promotion of breastfeeding might be understood as a moral sermon that cannot be put into practice; therefore the education of healthcare professionals should be considered the first priority before the education of the public.”* (Germany/Gynecologist/IBCLC)

**Fig. 2:**
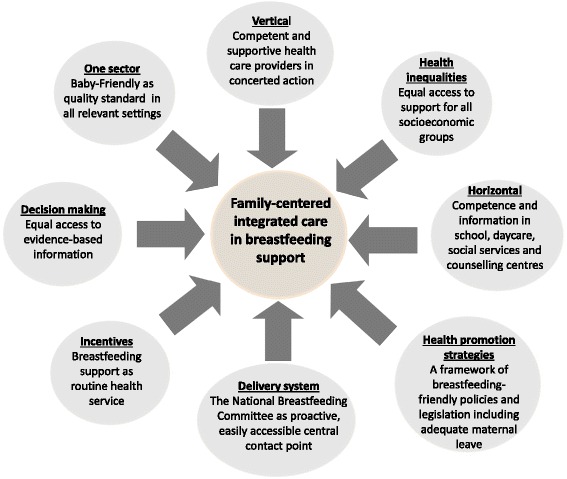
A family-centered model of integrated care in breastfeeding support

While the results of this study so far have focused on identifying barriers to integrated care in breastfeeding support, and strategies to overcome those deficits, Fig. 2 shows the patients’ perspective. This family-centered model is derived from all the survey results and abstracts them in the integrated care fields by filtering only the ideal outcomes of the suggested measures in terms of integrated care. These are reflected in a few keywords, relevant from a family perspective, thus omitting all the necessary measures “behind the scenes” in terms of policies and re-structuring of healthcare. Figure 2 aims to show the whole picture of an implemented framework of integrated care in breastfeeding support as a model of a patient-centered approach to facilitate successful breastfeeding.

## Discussion

### Integrated care within breastfeeding support

The importance of integrated care within breastfeeding support has not been extensively studied, with the exception of the Baby Friendly Initiative [43]. Yet, interventions including some form of collaboration among sectors or different types of health professionals or health professionals and volunteers often resulted in positive outcomes in terms of breastfeeding rates [30, 31, 44, 45] and significantly greater maternal satisfaction [32, 46], as recently described in the 2016 Lancet Breastfeeding Series.

Study participants considered the WHO/UNICEF Baby-Friendly Initiative important for setting and upholding quality standards for integrated breastfeeding support in key healthcare settings. In addition to these setting-related integrated care initiatives, this survey shows the need to integrate breastfeeding support within the wider healthcare system, including system incentives, which represents an important claim in the light of Kodner’s statement, that the structure and performance of integrated healthcare follows funding [2]. However, healthcare systems within industrialized countries currently seem to incentivize routine birth interventions [47] and supplementation with infant formula [48], representing practices known to decrease breastfeeding rates [49]. The lack of healthcare provider time, knowledge and skill in providing breastfeeding support, reported both in this survey and described in the literature as discontinuity of care [50], might be overcome by incentivizing breastfeeding support competence, quality and professional performance within healthcare systems [51].

Incentivizing breastfeeding support within healthcare might also be an adequate measure to counter the poor acknowledgement practitioners reported in this survey. System incentives promoting competence of breastfeeding support might counter educational deficits of healthcare providers. In this respect, respondents emphasized the education of physicians as vital, because of their elevated hierarchic position. Physicians in their role as primary caregivers also have an integrative function within healthcare, since primary care has a central role in integrating care within the healthcare system [52]. Moreover, respondents reported that the poor acknowledgement of breastfeeding support impairs collaboration within healthcare teams. Teamwork is compromised when this lack of recognition exists in combination with inferior work positions and high workload [53]. To facilitate vertically integrated care and integrated care within the maternity care sector, respondents call for implementing higher standards of training into the primary and continuing education of healthcare providers, as also reflected in the literature [54]. Given the WHO recommendation to continue breastfeeding up to two years and beyond [20], it is expected that mother-infant dyads will use various health services over several years from pregnancy to toddlerhood, which makes quality education of all healthcare providers within and beyond maternity healthcare mandatory, to achieve a continuum of care. The IBCLC qualification was generally considered the preferred form of training for those directly involved in maternal/infant healthcare (Table 2), except by respondents from Norway, Sweden, Belgium and Canada, perhaps because they operate in environments where the majority of health professionals receive a high standard of breastfeeding training [55].

The expansion of a milk bank network suggested by respondents, with donor milk recommended by WHO as third choice for infants before formula use [20], might also advance vertically integrated care within breastfeeding support, especially when implemented into national healthcare guidelines [56] and made available by the cooperation of healthcare providers from different care levels. This measure is also apt to reduce industrial influence and infant mortality [57, 58].

In our study, Norway was the exception in many regards, which can be explained given that Norway’s breastfeeding support system already fulfills many claims raised by study respondents. This includes adequate maternity leave, providing 80 % of the mother’s salary paid for 1 year, as one option to choose for mothers. Further, the nationwide and population-based expansion of the mother support group “Ammehjelpen”, developed from La Leche League principles [59], was supported by the Norwegian government, and has contributed, among other factors, to an increase of breastfeeding rates by over 40 % from 1968 to 1988 [60]. The standard Baby-Friendly accreditation of maternity services since the 1990s, combined with ongoing accreditation of neonatology wards and health centers appear to have been adequate to restructure healthcare towards improved integrated breastfeeding support, thus rebuilding their breastfeeding culture over more than four decades [61]. Another factor contributing to this success is the uninterrupted tradition of midwife-led births. With midwives representing the primary caregivers at birth, Norwegian hospitals have met most of the mother-friendly criteria then and now [62]. Moreover, by spreading the Baby-Friendly standard and establishing an agreement with the infant formula industry, Norway has given effect to the International Code of Marketing of Breast-milk Substitutes to a degree unmet by many other countries [63, 64]. Norway has also founded a National Resource Centre for Breastfeeding and a National Advisory Unit functioning as NBC, aimed at advancing breastfeeding protection and promotion, and preventing harmful commercial influence on healthcare, the public and research. Through systematic work to gain political as well as professional support, Norway has achieved and managed to sustain outstanding breastfeeding rates by means of a comprehensive, integrated approach [49, 60, 61], which is reflected in our study results.

When comparing this achievement with breastfeeding support on an international level, mother support groups rarely meet the high coverage achieved in Norway, currently amounting to about 1:36,000 counsellors per capita [e-mail communication of the first author with Ammehjelpen in October 2015], with the exception of La Leche League groups in New Zealand (about 1:31,000 counsellors per capita), followed by Luxembourg (about 1:37,000 counsellors per capita) and Canada (about 1:68,000 counsellors per capita) [25]. The literature describes the positive impact of mother support groups on breastfeeding rates [30, 65], making the promotion of peer support and enhanced collaboration of healthcare providers and volunteers, as suggested by survey participants, appear to be a good investment. For improved integrated care, peer support might also be included in healthcare pathways towards integrated care in breastfeeding support [66].

The worldwide implementation rate of ever designated Baby-Friendly Hospitals amounts to 27.5 %, with industrialized countries only scoring 8.5 % [67], showing that other countries fall short of Norwegian standards with 97 % accreditation [e-mail communication of the first author with the Norwegian Resource Center in January 2016]. To obtain a similar growth of breastfeeding rates, other countries with more inhabitants might need an even more comprehensive approach aimed at ingraining integrated care of breastfeeding support within and beyond key settings by using healthcare system incentives [2, 51], because the reported major failure of vertically integrated care cannot be resolved by setting-related approaches only.

Both midwives and practitioners in breastfeeding support play a decisive role in facilitating natural maternity processes, which represents common ground in the normative dimension of integrated care [68]. Their collaboration towards concerted action represents an essential component of integrated care in breastfeeding support, in the sense of a continuum of care. In this survey practitioners in breastfeeding support in addition to midwives describe an unsatisfactory integration of their profession into regular healthcare, which is reflected in current developments [69] and counter-strategies facilitating more natural births [70, 71]. In this respect, survey participants suggest that health insurance companies should take on the responsibility to incentivize natural birth and breastfeeding. towards sustainability of maternity healthcare, and on the other hand control and restrict over-medication and unnecessary interventions at birth [72–74], which reduce birthing choices for parents [47] and thus represent unfavorable routines for breastfeeding initiation [33, 62].

According to respondents, for parents, a shared decision on infant feeding is hindered by discontinuity of care, caused by healthcare providers poorly educated in breastfeeding support, inconsistent advice and poor societal support [50, 75, 76]. As a result, parents lack relevant facts for shared decision-making, and the practical how-to for successful breastfeeding enabling them to reach individual breastfeeding goals [77]. There are many examples from industrialized countries, where breastfeeding is initiated by between 70 % and 90 % of mothers, but breastfeeding rates drop rapidly within a few weeks [78, 79]. This indicates the intention of parents to breastfeed on one hand, and the failure of breastfeeding support on the other hand, thus reducing infant feeding choices for parents [31, 80]. The lack of consistency and continuity of care represents a decisive factor for discontinuation of breastfeeding, while the resource of peer support as social capital often remains unused [65, 66]. Access to competent breastfeeding support might even be more difficult for parents with lower socioeconomic status, potentially increasing health inequalities [81–85].

The overwhelming consensus of international practitioners in breastfeeding support is the lack of breastfeeding health promotion strategies, reflecting the half-heartedness of current policies on infant feeding in industrialized countries [86]. The most vulnerable population groups are often subject to formula industry marketing strategies, unhindered by governments or even with their support [87], as illustrated by the former version of the US Women, Infant and Children program [88] and the current lobbyist activities in Canada [89]. Ineffective policies can cause more damage than no policies, because they prevent progress by pretending that measures have already been taken [86]. According to the results of this survey, building political consensus and a follow-up of health promotion strategies is essential to effectively implement integrated care within breastfeeding support.

As reports from different countries show, National Breastfeeding Committees may noticeably impact breastfeeding rates, an important health outcome, when empowered and supported by their governments to take on the National leadership in breastfeeding protection, promotion and support [90–92]. This includes spreading the Baby-Friendly Initiative and constructing policies effectively to fulfill their original tasks [16]. According to respondents, only about half of the existing policies to protect and promote breastfeeding within this sample work efficiently and make an impact. NBCs lack funds, power and influence. This shortcoming of policies might be due to industrial influence and their non-transparent strategies [86–89], and a lack of Code implementation [19, 57], preventing progress towards integrated breastfeeding support.

In our study, respondents saw National Breastfeeding Committees playing a key role in breastfeeding protection and promotion as a delivery system, calling upon governments to create this institution and provide financial support for the many varied and important functions of this authoritative body. The newly defined tasks of the National Breastfeeding Committee by respondents may assist governments in establishing integrated care in breastfeeding support, steering towards breastfeeding-friendliness of society and cost-efficiency, prevention and sustainability within healthcare [93, 94]. Valentijn et al. [68] describe normative and functional integration at three levels of healthcare: micro (clinical), meso (healthcare professional and organizational) and macro (system) integration. In terms of breastfeeding support this means that the NBC should ensure normative integration towards breastfeeding-friendliness within and beyond healthcare, providing seamless connectivity across all three levels in the sense of functional integration. Thus the NBC faces a complex challenge in promoting society-wide support for breastfeeding.

The task for all healthcare systems to overcome unfavorable routines, structures, legislation and quality gaps [17, 18, 30, 31, 62, 74, 77, 78, 95]; and develop towards integrated care in breastfeeding support and sustainablity, implies a long-lasting process. Even with active support from the government, Norway took several decades to establish improved quality of breastfeeding support within healthcare and society as an ongoing process, including the current accreditation of communities as Baby-Friendly. On the other hand, the half-hearted policies and reduction of midwifery services in Germany, contribute to the disintegration of breastfeeding support [69, 86], indicating that on a global level there is not only progress, but also regression of integrated care in breastfeeding support. This backlash and slow progress is reflected in the overdue implementation, since the 1980s, of international public health nutrition policy initiatives [13–20]. Against this background, this study represents a highly topical approach to establish healthcare systems that are geared to providing integrated care to support breastfeeding mothers.

### Strengths and limitations

Even though participants in this study represent a convenience sample of self-selected practitioners in breastfeeding support, the majority were from high income European countries, their knowledge about the obstacles to integrated breastfeeding support, which they experience in their everyday work environment, is considerable and worth noting.

This survey investigates for the first time the opinions of international practitioners in breastfeeding support, and puts integrated care in breastfeeding support in perspective. Respondents described aspects of integrated care within many different healthcare settings, covering a broad spectrum of topics, enabling us to provide a comprehensive overview of its relevant components. Moreover, an evaluation of National Breastfeeding Committees’ performance, from practitioners’ perspective, including their expectations, has not been explored, either. Agreement of opinions among international participants, e.g. on the major importance of integrated care and health policies to facilitate effective breastfeeding support, gives additional strength to the findings. These might be useful for policy makers to further breastfeeding protection, promotion and support on a national or global level. Further research might focus on the implementation of these suggested measures and policies, and on the evaluation of their results in terms of breastfeeding rates.

## Conclusions

The task of providing integrated care in breastfeeding support to facilitate breastfeeding initiation and sustainment is a challenging one, requiring a re-structuring of the healthcare system. The new structure would involve integrating lactation consulting as a profession, educating all healthcare professionals in breastfeeding support, creating system incentives for natural birth and breastfeeding, and implementing quality standards in key healthcare settings, such as the Mother- and Baby-Friendly accreditation. A continuum of care, involving the cooperation of competent healthcare providers, is vital for families to experience shared decision-making regarding infant feeding and the how-to of successful breastfeeding, independent of their socioeconomic background. This comprehensive task cannot be accomplished successfully without strong political consensus and a clear health policy to protect, promote and support breastfeeding as a sustainable resource of public health, which might be coordinated by an empowered National Breastfeeding Committee.

## Additional files

Additional file 1: The failure of vertical integration of breastfeeding support, according to the categorization of open-ended responses. (DOCX 25 kb) Additional file 2: The failure of health promotion strategies, including suggestions for improvement, according to the categorization of open-ended responses. (DOCX 24 kb)

## Abbreviations

ABA: Australian Breastfeeding Association
ABM: Academy of Breastfeeding Medicine
AFS: Arbeitsgemeinschaft Freier Stillgruppen [Work group of free breastfeeding support groups], represented in Germany and Austria
BFCI: Baby-Friendly Community Initiative
BFHI: Baby-Friendly Hospital Initiative
Code: International Code of Marketing of Breast-Milk Substitutes
ELACTA: European Lactation Consultants Alliance
FABM: Fellow of the Academy of Breastfeeding Medicine
IBCLC: International Board Certified Lactation Consultant
IBFAN: International Baby Food Action Network
ILCA: International Lactation Consultant Association
LC: Lactation Consultant
LLL: La Leche League
MFHI: Mother-Friendly Hospital Initiative
NBC: National Breastfeeding Committee
UNICEF: United Nations International Children’s Emergency Fund
VELB: Verband Europäischer LakationsberaterInnen [Association of European Lactation Consultants]
WHO: World Health Organization
WIC: Women, Infants, and **C**hildren (US Supplemental Nutrition Program)
