# Supplementary material for: Towards integrated care in breastfeeding support: a cross-sectional survey of practitioners’ perspectives
Source: Int Breastfeed J. 2016 Jun 3;11:15. doi: 10.1186/s13006-016-0072-y (PMC4891910; doi:10.1186/s13006-016-0072-y)
Supplement: Additional file 1: — The failure of vertical integration of breastfeeding support, according to the categorization of open-ended responses. (DOCX 25 kb) [file 13006_2016_72_MOESM1_ESM.docx]

Additional File 1: The failure of vertical integration of breastfeeding support, according to the categorization of open-ended responses

| *Vertical integrated care category* | *Sub-category* | *Aspects mentioned* | *Quotations*  *(Residency/ Profession /Qualification)* |
| --- | --- | --- | --- |
| Lack of evidence-based breastfeeding knowledge amongst healthcare providers  **21.2% / n = 64** | Lack of skills, knowledge and expertise  **3.6% / n = 11** | A general lack of healthcare professionals’ knowledge, e.g. physicians/pediatricians/gynecologists/nutritionists, resulting in a poor support for breastfeeding mothers, unnecessary suffering of the mother-baby dyad and unnecessary or routine supplementation of infant formula, undermining milk supply, mothers’ confidence and breastfeeding success | *“There is no progress in the implementation of breastfeeding knowledge”* (Austria/ Gynecologist/IBCLC) |
|  | Suggestions to counteract this lack of education  **12.9% / n = 39** | Standard education for all healthcare providers  Great need for education in medical schools  Standard training for physicians contributes to promoting breastfeeding and has the potential to change people’s attitudes towards breastfeeding | *“The focus should be on the education of physicians in breastfeeding support”* (Austria/Free-lance LC/ IBCLC + LLL) |
|  | Continuing education of healthcare providers in breastfeeding support is lacking  **4.6% / n = 14** | Continuing training and communication of up-to-date and evidence-based information to healthcare providers as a standard, funded by employers  The National Breastfeeding Committee should take on an active role, communicate its purpose and goals to schools of nursing and hospitals, filter research results for healthcare professionals and provide them relevant and up-to-date information,  promote breastfeeding by exchange of knowledge | *“A network of education centers in breastfeeding support for healthcare providers should be established”* (Hungary/ Pediatrician/IBCLC) |
| Failure of integrated care in breastfeeding support within healthcare  **45.5% / n = 137** | Description of the general failure of healthcare in providing integrated care in breastfeeding support  **6.3% / n = 19** | Lacking support and acceptance of the lactation specialty by other healthcare providers/peers/work environment/team/ward  No concerted action by healthcare providers for breastfeeding support  No support of exclusive breastfeeding by interdisciplinary team of healthcare providers  Healthcare providers counteract breastfeeding support  Mothers should benefit from integrated care and enjoy continuous breastfeeding support throughout pregnancy, birth and post-partum | *“Sometimes I am frustrated because other healthcare providers destroy my work out of ignorance”* (Germany/ Midwife/IBCLC)  *“Poor interdisciplinary cooperation”*  Belgium/Midwife/IBCLC)  *“I feel like I am swimming against a very strong current”* (Israel/LC in hospital/IBCLC + LLL) |
|  | Suggestions to improve integrated care in breastfeeding support in general  **6.6% / n = 20** | Better coordination and networking  Regional network of breastfeeding promoting unions: Round table of midwives, hospitals, doctors  More interdisciplinary cooperation  A more correct implementation of standards  Earlier rals from other healthcare providers are necessary | *“The awareness level of lactation consulting possibilities is too low”* (Germany/General Nurse/ IBCLC + LLL) |
|  | Failure of cooperation with hospitals or after discharge  **2.9% / n = 9** | Lack of cooperation with and support from hospital staff, e.g. on maternity ward or neonatology  Breastfeeding initiation inside the hospital fails  A lack of integrated care after hospital discharge | *“More cooperation with lactation consultants within and outside of the hospital is needed”* (Switzerland/Midwife/ Healthcare Provider) |
|  | Failure of cooperation with physicians  **10.2% / n = 31** | Obstacles: disregarding attitudes/arrogance and ignorance: a lack of interest/a lack of support or acknowledgement by physicians/or by the head physician, in hospitals or private practice  Physicians do not recognize the importance of breastfeeding for child health, sometimes implying that formula was just as good as human milk  The cooperation of physicians and lactation consultants needs improvement | *“Interventions at childbirth and artificial infant feeding disempower the family”* (Italy/ Pediatrician/IBCLC) |
|  | Failure of cooperation with pediatricians  **2.9% / n = 9** | Non-supportive attitudes / ignorance / disrespect/ a substantial lack of support of lactation consulting by pediatricians  Pediatricians supplement when not necessary, not considering the importance of breastfeeding | *“Pediatricians lack of appreciation of breastfeeding: „Nice-to-have, but not really necessary“* (Austria/ Pediatrician/IBCLC) |
|  | Failure of cooperation with gynecologists and obstetricians  **2.9% / n = 9** | The increase of Caesarean sections and interventions in childbirth have a negative impact on initiation and duration of breastfeeding Gynecologists and obstetricians lack of interest in breastfeeding support | *“Health insurance companies should control and restrict interventions at childbirth”* (USA/ Maternity Nurse/IBCLC) |
|  | Failure of cooperation with midwives  **1.6% / n = 5** | Midwives counteract lactation consulting  Midwives do not promote breastfeeding | *“An improved cooperation with midwives is needed”* (Germany/ General Nurse/ IBCLC+LLL) |
|  | Lack of recognition by colleagues  **4.9% / n = 15** | Lacking support from colleagues / the team  Disregarding / averse attitudes of colleagues  Colleagues not promoting breastfeeding  Colleagues undermining lactation consultant’s work during her absence | *“Many colleagues refuse to re-think their attitudes to make a change, in spite of the challenging task ahead”* (Austria/ LC in hospital/ IBCLC) |
|  | Lack of support from superiors **6.6% / n = 20** | Support /acknowledgement / appreciation from employer / manager / supervisor / hospital administration is lacking  Financial support for continuing education is lacking | *“A lack of support and financial recognition by employer, e.g. I am attending conferences at my own expense”* (Canada/ Dental Hygienist+LC/ IBCLC+LLL |
| Lack of cooperation with research  **11.2 / n = 34** | Suggestions to improve the cooperation with researchers  **4.6% / n = 14** | Practice-oriented research  Intense cooperation of science and practice  Research should be designed to back up health professionals  A constant information flow should enable the implementation of new insights by healthcare providers  Carry out more studies together with lactation consultants | *“Researchers should translate their results into clear and understandable recommendations for practitioners”* (Australia/ Midwife/ IBCLC) |
|  | Lack of researchers' knowledge on breastfeeding  **6.6% / n = 20** | Lack of acknowledgement of breastfeeding as the norm for infant feeding  Researchers are lacking of basic knowledge on breastfeeding, not recognizing the well-known facts  Accurate monitoring of the breastfeeding rates is lacking  More knowledge and understanding and more appreciation of the value of breastfeeding is needed | *“Breastfeeding should be considered as the norm for infant feeding in research”* (Netherlands/ Maternity Nurse/ IBCLC) |
| Integrate voluntary breastfeeding support  **6.3% / n = 19** |  | Voluntary breastfeeding counsellors want to be taken seriously as experts  More promotion of mother support groups including financial support  Improve the cooperation of voluntary and professional breastfeeding counsellors  Create more new breastfeeding support groups  Reach more mothers | *“Enable easy access to mother support groups”* (Netherlands/ General Practitioner/ Healthcare Provider) |
| The creation of a human milk bank network is important  **4.6% / n = 14** |  | The immunological power of human milk is important  Breastfeeding will be viewed as the healthiest choice for both mothers and infants within the next 15-20 years  Mothers will gain self-confidence, autonomy and independence of the industry by breastfeeding, within the next 15-20 years  Human milk will be the only milk for human babies within the next 15-20 years | *“The insight that human milk is the best choice for human babies will be established within the next 15-20 years”* (Germany/ Bookseller/ LLL) |
| **Total: 268** |  |  |  |
