# Supplementary material for: Towards integrated care in breastfeeding support: a cross-sectional survey of practitioners’ perspectives
Source: Int Breastfeed J. 2016 Jun 3;11:15. doi: 10.1186/s13006-016-0072-y (PMC4891910; doi:10.1186/s13006-016-0072-y)
Supplement: Additional file 2: — The failure of health promotion strategies, including suggestions for improvement, according to the categorization of open-ended responses. (DOCX 24 kb) [file 13006_2016_72_MOESM2_ESM.docx]

Additional File 2: The failure of health promotion strategies, including suggestions for improvement, according to the categorization of open-ended responses

| *Category* | *Aspects mentioned within the category* | *Quotations*  *(Residency/Profession/Qualification)* |
| --- | --- | --- |
| Health policies are lacking  **21.9% / n = 66** | Lack of a clear commitment and breastfeeding-friendliness of governments  Lack of political activity and recognition of breastfeeding’s importance  Lack of efforts to increase the breastfeeding rate  Lack of consumer protection  Lack of NBCs’ foundation in several countries  Lack of governmental and NBC support | “There are only cost cutting measures instead of official political support” (Germany/ Maternity Nurse/IBCLC) |
| Health policies are failing  **5.6% / n = 17** | Non-supportive/non-effective health policies  Women are disempowered by not facilitating breastfeeding | “Policies should have teeth“, they must be enforced and implemented” (Germany/General Practitioner/FABM+IBCLC+LLL) |
| Implement policies to protect and promote breastfeeding  **18.9% / n = 57** | Adhere to and enforce WHO/UNICEF recommendations  Implement evidence-based breastfeeding standards in all domains  Develop nation-wide activities  Reach decision makers, raise awareness and efficiency  Promote health by promoting breastfeeding  Unequivocal support and protection of breastfeeding  Integration of breastfeeding promotion into national health policy  Plan and coordinate a national policy and implement it with political support | “Health policies should take on the responsibility to protect and promote breastfeeding.”(Austria/ Gynecologist/IBCLC) |
| Counteract the conflict of interest in health policies  **2.3% / n = 7** | Act for children and not for profit  Abandon the economic interests of the pharmaceutical industry  Promote health and prevention in opposition to industry lobbyists  Base health policies on evidence rather than on economics and tradition  Family-based policies instead of commercial-interest policies | “Economic interests should no longer be the priority in health policies” (Germany/Bank clerk/ LLL) |
|  |  |  |
| Establish breastfeeding promotion as preventive measure  **8.3% / n = 25** | Acknowledge breastfeeding and human milk’s preventive effect against illness, violence and child abuse, and as health promoting factor including its benefits for physical and mental health, long-term effects, and risks and side effects of substitutes  Focus research on the preventive effect of breastfeeding  Support the reproductive health continuum | “More focus of health policies on early prevention, nutrition policy and ethics” (Germany/ Gynecologist/IBCLC) |
| The National Breastfeeding Committee should have an impact on health policies and lobby for breastfeeding  **6.3% / n = 19** | The National Breastfeeding Committee should have a greater political impact on the healthcare sector and advocate the economic benefits of breastfeeding for the healthcare system  The National Breastfeeding Committee should have more political weight and implement its results  Lobbying activities at the health department for breastfeeding are needed  Present a united front for breastfeeding advocacy | “The National Breastfeeding Committee should take on the national leadership in promoting breastfeeding” (Australia/Free-lance Lactation Consultant/ IBCLC) |
| Enable the cooperation of breastfeeding advocates  **6.9% / n = 21** | Facilitate communication and skill-building of state breastfeeding coalitions  Organize projects to promote breastfeeding  Exchange knowledge  Cooperate with health politicians  Coordinate all breastfeeding activities | “Set National policy, revise policies regularly, regular meetings” (Italy/ Pediatrician + Researcher/ Healthcare Provider) |
| Develop standards, recommendations, guidelines and follow-up  **5.6% / n = 17** | Create National guidelines/standards  Control and evaluation of measures is needed  Develop nation-wide recommendations  Clear standards and efficiency is needed, like in Northern European countries  Set clear detailed policy on breastfeeding promotion  Productivity and a follow-up of policies is needed | “The National Breastfeeding Committee should release recommendations for health policies” (Germany/Homemaker/ LLL) |
| Take action on different political levels  **6.3% / n = 19** | Bad German family policies impair breastfeeding  More breastfeeding promotion (e.g. by mother support groups) is needed in developing countries / cities / communities  Leverage governmental support on each level | “In Australia changes have been implemented by individual states, but it requires a complete national level with policy”  (Australia/Nurse/IBCLC+ABA) |
| Create a favorable legislation for breastfeeding  **6.6% / n = 20** | The National Breastfeeding Committee should advise the government on legislation to support breastfeeding and advocate the rights of breastfeeding mothers  Counteract the requirements of modern work that don't allow mothers to combine work and breastfeeding  Implement adequate maternal leave and other breastfeeding-friendly laws  Employers should provide facilities to pump milk | “Enforce laws to promote, protect and support breastfeeding as the norm” (Italy/Obstetrician-Gynecologist/IBCLC) |
| Implement the International Code for the marketing of breast milk substitutes as a law **5.6% / n = 17** | Prohibit the advertisement for substitutes, also for babies over 6 months of age  Uncover commercial interests and scandals | “Breastfeeding promotion will not be successful in the face of continued strong advertisements for substitutes” (Germany/Pediatric Nurse/IBCLC) |
| Control, monitoring and penalty for Code violations  **6.3% / n = 19** | Uphold the Code  Strict control and monitoring of implemented rules | “The protection of the mother-baby dyad is lacking” (USA/Obstetrician/IBCLC) |
| Promote high-quality and ethically sound research on infant feeding, independent of commercial interests  **22.5% / n = 68** | Adhere to the International Code for the Marketing of Breast Milk Substitutes in research  Promote research independent of the substitute producing industry  Disable misuse of research and lack of breastfeeding knowledge in research  More research results, also from Germany  More new insights in breastfeeding  Evidence-based studies and hard data to support breastfeeding  Good quality of studies, broad studies  More interdisciplinary research  Set priorities for essential research  Focus research on the promotion of breastfeeding  Up-to-date research results on breastfeeding promotion | “Research for health and not for profit” (Netherlands/Neonatal Nurse/IBCLC)  “Increase independent funding and research opportunities”  (USA/Pediatrician/IBCLC)  “Independent research, not beholden to commercial interests, focused on health outcomes and prevention”  (Germany/Psychotherapist, LC/ IBCLC+LLL) |
| Improve the cooperation of health policies and research  **3.3% / n = 10** | A close collaboration of health policies with health sciences and the National Breastfeeding Committee  More interdisciplinary research  Health policies should be based on the current state of the art of research | “Mainstream the role of breastfeeding in every aspect of public health where it is relevant” (USA/Maternity Nurse/IBCLC) |
| Health policies should enable research implementation  **5.3% / n = 16** | Research on how-to support families to breastfeed for infant health  Statistics on breastfeeding should be standard  Publication of the hard data for political action  Improve the recommendations for medication and therapy of breastfeeding mothers | “Incorporate research results into breastfeeding activities” (Uganda/ Social Scientist/Breastfeeding Mother) |
| Lacking support and funding for breastfeeding protection, promotion and support should be gained  **13.9% / n = 42** | Gain politicians as powerful supporters for breastfeeding  Gain support of health insurances  Gain governmental support  Gain financial support to promote breastfeeding and to implement existing programs | “Progress of breastfeeding protection and promotion is only possible with major investments” (Germany/Pediatric Nurse/IBCLC) |
| Health policies should facilitate a patient-centered approach  **2.6% / n =8** | Implement optimal conditions for breastfeeding mothers  More understanding of breastfeeding mothers is needed  Create an integrated breastfeeding policy for women | “Implement a child-, family- breastfeeding- and mother-friendly policy” (Switzerland/Midwife/IBCLC) |
| Prioritize breastfeeding protection and promotion  **1.9% / n = 6** | More activism, change policies, so that breastfeeding protection and promotion become a priority | “Breastfeeding should be promoted as a priority, while the far ranging consequences of not breastfeeding should be pointed out” (Germany/ Teacher/LLL) |
| **Total: 454** |  |  |
